# 

**Published:** 1991-06

**Authors:** 


					W1 WE403
V.106 NO.2 1S9L
C-01 SEQ: SR006S7S8
Ti: WEST OF ENGLAND MEDICAL
JOURNAL
Volume 106(ii)
June 1991
The Changing Face of Bristol Medicine
Four Hunterian Lectures
Screening for Diseases
Towards Better Discharge Summaries
Perforation of a Peptic Ulcer into the Left Ventricle
Non-Immune Hydrops
Recurrent Radial Head Dislocation
Book Reviews
Abstracts of Meetings
I??EMISBL? IBEHSTeiL M?M<D<Q>=OTmUK(|lI(DAIL JOUBNAIL
ISSN: 0960 6440 NLM Code B5Q

				

